# Hyperprolactinemia in Older Adults: A Narrative Review of Distinct Clinical Challenges and Management Strategies

**DOI:** 10.7759/cureus.103385

**Published:** 2026-02-10

**Authors:** Andres F Quimbayo-Cifuentes

**Affiliations:** 1 Internal Medicine and Geriatrics, Centros Medicos Colsanitas SAS, Armenia, COL

**Keywords:** aged, comorbidity, diagnosis, hyperprolactinemia, pituitary diseases, prolactin, therapeutics

## Abstract

Hyperprolactinemia in older adults represents a significant diagnostic and therapeutic challenge, as its manifestations can often be masked by symptoms typically associated with normal aging. Unlike in younger populations, the primary cause of this condition in the elderly is the use of medications, such as antipsychotics, antiemetics, and prokinetics. The clinical presentation of excessive prolactin production in the elderly varies significantly, and the diagnostic approach requires a high index of clinical suspicion and a thorough review of potentially involved medications. Management must be individualized, carefully selecting candidates for dopamine agonist therapy or, in cases of pituitary adenomas, surgical or radiotherapeutic management. This review synthesizes the evidence on these atypical presentations and the nuances of management tailored to the older adult.

## Introduction and background

Prolactin (PRL) is a peptide hormone primarily secreted by the lactotroph cells of the anterior pituitary gland, with its primary function being the facilitation of lactation [1]. PRL secretion is regulated by dopamine-mediated inhibition, which acts on D2 receptors of the lactotroph cells [2].

Hyperprolactinemia (HPRL) is biochemically defined by serum PRL levels exceeding the upper limit of normal, with widely accepted consensus thresholds of >25 ng/mL for women and >20 ng/mL for men [3]. It is essential that the initial measurement is performed while minimizing venipuncture-related stress; furthermore, screening for macroprolactinemia (MPRL) should be considered, as macroprolactin-a biologically inactive polymeric form-can produce falsely elevated results [1].

Although prolactinomas are the most common subtype of functional pituitary adenoma [4], the prevalence of clinically relevant cases decreases in patients older than 65 years [5]. In the elderly, the clinical presentation is frequently atypical and insidious; manifestations such as sexual dysfunction, fatigue, and weakness are often non-specific and erroneously attributed to the normal aging process [6,7]. This combination of factors leads to significant delays in the diagnosis of HPRL. Furthermore, the condition frequently has a drug-induced origin in this population due to the high prevalence of polypharmacy, with antipsychotics, antidepressants, and prokinetics being common causative agents via D2 receptor blockade [8,9]. A potential long-term risk of chronic elevation is the development of osteoporosis and fragility fractures [4]. Consequently, treatment with dopamine agonists must be approached cautiously to avoid complications associated with their use in older adults [9,10]. The objective of this narrative review is to synthesize existing evidence regarding the etiology, clinical presentation, diagnostic approach, and therapeutic options for HPRL tailored to the particularities of the geriatric population.

## Review

PRL homeostasis and aging

The analysis of serum PRL levels requires an understanding of age-induced physiological changes, which exhibit sex-specific differences [11]. In healthy women, there is a documented decline in circulating PRL levels following menopause, reflecting reduced estrogenic stimulation; this decrease can be so significant that pre-existing HPRL may normalize spontaneously [12]. In healthy older men, the relationship with PRL is more complex. While some cross-sectional studies suggest that serum levels may remain relatively stable or show only a minimal decrease with age, a significant decline in secretory dynamics has been documented [13]. This includes an approximately 2.5-fold decrease in the mass of PRL released per pulsatile episode and a twofold reduction in the basal secretion rate [14].

Etiological landscape in the older adult

Non-physiological HPRL in the elderly may arise from pharmacological agents, pathological conditions such as prolactinomas, or systemic disorders [5,13,15]. However, the most frequent cause of non-tumoral HPRL is pharmacological [1], a factor especially relevant in the older adult, as this population often presents with multiple comorbidities and consumes various drugs that can elevate PRL levels [9].

The primary pharmacological mechanism is the blockade of dopamine D2 receptors in the anterior pituitary, which removes the tonic dopaminergic inhibition of PRL secretion [9-16]. Antipsychotics (neuroleptics) are the most common agents causing iatrogenic HPRL, and this elevation can be significant, reaching up to 90% in women and 70% in men who use them [1,9,17]. Typical (first-generation) antipsychotics, along with certain atypical antipsychotics such as risperidone, paliperidone, and amisulpride, are known to cause the most marked elevations [9-18]. Indeed, PRL levels in patients taking classic antipsychotics are significantly higher than in those taking second-generation antipsychotics [19]. In addition to antipsychotics, other pharmacological groups contribute to HPRL in elderly patients. These include medications acting through serotonergic pathways, such as antidepressants, as well as prokinetics and antiemetics that act as D2 receptor antagonists, such as metoclopramide and domperidone [20,21]. Other frequent agents include opioids and certain antihypertensives, such as verapamil or alpha-methyldopa [22,23]. In general, the concomitant use of several drugs with hyperprolactinemic potential is associated with higher PRL levels.

While HPRL in the elderly may also be due to pituitary pathologies (such as prolactinomas) or non-secretory tumors causing the "stalk effect," iatrogenic HPRL imposes a crucial diagnostic and therapeutic challenge in this population [1-9]. Common systemic causes in old age play a vital role. Chronic kidney disease (CKD) is a well-recognized cause of moderate HPRL. This condition is present in approximately one-third of patients with renal disease and is due to a decrease in the renal clearance of PRL [24]. Additionally, untreated primary hypothyroidism is another significant systemic cause, as the excess of thyrotropin-releasing hormone (TRH) directly stimulates lactotroph cells [1-25]. In cases of long-standing duration and lack of adequate treatment, this chronic stimulation can even lead to pituitary hyperplasia, mimicking a pituitary adenoma [1].

Regarding pituitary pathology and stalk lesions, HPRL can be induced by the stalk effect [4]. This phenomenon occurs when a non-secretory sellar mass (the majority of pituitary tumors in the elderly) compresses the pituitary stalk, blocking the flow of dopamine-the primary PRL inhibitor-from the hypothalamus to the pituitary gland [4,26]. HPRL due to the stalk effect usually generates lower PRL levels than those observed in secretory prolactinomas, although PRL levels exceeding approximately 200 μg/L (or 250 μg/L in some sources) strongly suggest a true prolactinoma [1,4]. In addition to adenomas, the possibility of pituitary metastases should be considered in the elderly population; these are more frequent in this group and usually originate from breast, lung, or prostate cancers [27]. Metastases may present as sellar masses causing HPRL via the stalk effect or, more rarely, through ectopic PRL secretion [28,29].

Finally, it is crucial to recognize MPRL, a laboratory condition that can lead to misdiagnosis and unnecessary treatment. MPRL is due to the presence of macroprolactin (high molecular weight PRL complexes) in the blood, which are biologically inactive but cross-react with laboratory immunoassays, resulting in falsely elevated serum PRL levels [30]. It is estimated that 5% to 25% of results indicating HPRL are falsely elevated due to MPRL [31]. If MPRL is not recognized, this apparent HPRL can cause clinical confusion, unnecessary investigations (such as MRIs or surgery), and a waste of healthcare resources [31,32]. MPRL cannot be distinguished from true HPRL based solely on clinical presentation; therefore, systematic screening using macroprolactin precipitation with polyethylene glycol (PEG) is advised in all cases of HPRL [1,31].

Atypical clinical manifestations and clinical sequelae

The presentation of HPRL in elderly populations is frequently subtle. Classic signs, such as galactorrhea and amenorrhea, are less common or absent [33]. In postmenopausal women, amenorrhea is no longer a distinctive sign, and galactorrhea is rare [34]. In the absence of these indicators, the diagnosis of prolactinomas in postmenopausal women is often based on mass effect symptoms [12-34].

HPRL induces a state of hypogonadotropic hypogonadism, leading to a reduction in sex steroids [35]. This hypogonadism contributes to common complaints erroneously attributed to aging [33]. Decreased libido is a frequent manifestation in both men and women, as excess PRL exerts a direct adverse effect on sexual desire, independent of sex hormone levels [5]. In men, this hypogonadal state additionally manifests as erectile dysfunction [4].

Prolonged HPRL disrupts bone homeostasis primarily by inhibiting the pulsatile release of gonadotropin-releasing hormone (GnRH), which induces a state of hypogonadotropic hypogonadism and a subsequent deficit in sex steroids [36]. This chronic deficiency leads to reduced bone mineral density, resulting in osteopenia and osteoporosis [37,38]. This risk is particularly critical in older patients with antipsychotic-induced HPRL [9]. A high incidence of vertebral fractures and low bone mineral density has been documented in postmenopausal women with HPRL [34]. In a cohort of elderly men (≥65 years) with prolactinomas, the vast majority of evaluated patients presented with osteopenia or osteoporosis [8].

HPRL and associated hypopituitarism may manifest with non-specific symptoms such as fatigue, malaise, and mood changes (anxiety, depression) [33]. PRL participates in the regulation of neurogenesis in the hippocampus, suggesting a possible influence on cognitive abilities [39]. HPRL has been associated with potential cognitive impairment, affecting working memory and processing speed [40]. Improvement in cognitive function has been observed following the normalization of PRL with dopamine agonists, although specific evidence in older adults is limited [40].

Diagnostic workup

The diagnostic process for HPRL in the elderly must be methodical, establishing the condition through a single measurement of serum PRL while minimizing venipuncture-related stress. This precaution is essential because acute physical or emotional stress can trigger a transient physiological increase in secretion from the anterior pituitary that may lead to a false diagnosis [1,41]. The most important initial diagnostic intervention is a thorough medication reconciliation, due to the high prevalence of drug-induced HPRL [1,19]. If suspected, drug discontinuation for a minimum of three days-or a period tailored to the medication's specific half-life-or substitution is suggested (if clinically safe and in consultation with a specialist), followed by a new PRL measurement. If PRL levels are extremely high (10 times the upper limit of normal or more), they are unlikely to be due to drugs alone, and a pituitary MRI should be considered [1,5].

The reliability of PRL measurement must also consider critical analytical interferences [31]. MPRL, a high-molecular-weight form bound to IgG antibodies, lacks in vivo biological activity but reacts in most immunoassays. This artifact is a frequent cause of false HPRL, affecting up to 25% of results [30-32]. Although the global prevalence of MPRL does not show significant variation by age, one study found its incidence in women with HPRL increased progressively with age, reaching 42% in those over 45 [42,43]. Failure to detect it can lead to unnecessary investigations and inappropriate treatments. Another important diagnostic artifact is the hook effect, which occurs when extremely high PRL concentrations saturate the assay antibodies, resulting in a falsely low measurement [1,44]. Since macroprolactinomas, though rare, are more frequent in this population, guidelines recommend serial dilution of samples when there is a discrepancy between a large pituitary tumor and a minimally elevated PRL level [1,45].

Once iatrogenic causes and analytical artifacts have been systematically excluded, a broader assessment is necessary. Given that large tumors can manifest as hypopituitarism, a complete hormonal evaluation (morning cortisol, thyroid-stimulating hormone, free T4, insulin-like growth factor 1, luteinizing hormone, follicle-stimulating hormone, and sex hormones) is crucial [42]. High-resolution MRI of the sella turcica is the preferred imaging technique [1,5]. The indication is similar to that for young adults: upon suspicion of a prolactinoma or when HPRL is not explained by drugs [33]. In the elderly, adenomas often present as macroadenomas [33]. MRI is essential to differentiate the etiology, allowing for the distinction between drug-induced HPRL and a pituitary mass [1]. It is also crucial for evaluating mass effect, especially when neurological symptoms such as headaches or visual disturbances are present [33]. Finally, imaging allows for the exclusion of the stalk effect, which occurs when non-secretory sellar masses compress the pituitary stalk, interfering with dopaminergic inhibition [4].

Therapeutic strategies and management nuances in the elderly

The management of HPRL in patients older than 65 requires an individualized approach [27]. Older adults tend to accumulate risk factors for the adverse effects of HPRL, such as osteoporosis, which can lead to functional loss [17]. Unless symptoms of HPRL are distressing, monitoring without intervention may be the best strategy for frail older individuals [9,17].

In cases of antipsychotic-induced HPRL, strategies should prioritize stabilizing the mental health condition that led to the use of these medications. If HPRL is asymptomatic, the recommendation is not to treat and to perform watchful waiting with periodic PRL controls [17]. If symptomatic, the first option is to discontinue or substitute the drug for one with a lower effect on PRL (e.g., aripiprazole or clozapine), if clinically feasible [1]. The addition of dopamine agonists is controversial due to the risk of exacerbating psychosis, although the use of aripiprazole as an adjuvant has proven to be a safe strategy for reducing HPRL induced by other antipsychotics [9].

For the management of symptomatic prolactinoma, dopamine agonist therapy (primarily cabergoline) is the first line at all ages [1,4]. Cabergoline is preferred over bromocriptine due to its superior efficacy and tolerability [1]. Available evidence, though limited, suggests that older men (≥65 years) with prolactinomas respond to dopamine agonist treatment as well as or even better than their younger counterparts, achieving PRL normalization, tumor reduction, and clinical improvement in nearly all patients [8].

It is fundamental to initiate dopamine agonists at low doses and titrate gradually in the elderly due to the increased risk of adverse effects [5]. Orthostatic hypotension is a significant concern [46]. Cabergoline can cause neuropsychiatric side effects, including psychosis [47]. Although the risk of cardiac valvulopathy associated with cabergoline is low with the standard doses used in endocrinology (0.5-2.0 mg/week) and does not appear to be higher than the baseline risk in the general population, the prevalence of valvulopathy increases with age; thus, surveillance should be maintained [10].

In postmenopausal women with microprolactinomas, withdrawing existing dopamine agonist therapy or opting for a watchful waiting approach without initiating treatment may be considered, given the physiological decline in PRL levels following the loss of estrogenic stimulation [4,34]. However, in cases of macroprolactinomas, caution is advised when considering therapy withdrawal due to the potential, though rare, risk of tumor growth [34].

Transsphenoidal surgery and radiotherapy are reserved for specific scenarios, similar to the younger population, but with stricter consideration of potential perioperative risks [33]. Surgery is indicated for patients with intolerance or resistance to dopamine agonists and in patients with pituitary apoplexy [4]. Literature has demonstrated the relative safety of endoscopic transsphenoidal surgery in selected older adults, suggesting that age alone is not a contraindication [26]. Radiotherapy is a second- or third-line therapy for recurrent or resistant tumors but requires indefinite surveillance due to the high risk of hypopituitarism [4].

## Conclusions

HPRL in the elderly represents a significant diagnostic challenge due to its atypical clinical presentation. Classic signs are infrequent, and diagnosis is often delayed, based on mass effect symptoms or sequelae such as hypogonadism and osteoporosis, symptoms frequently masked by the aging process. Pharmacological etiology, driven by polypharmacy, is the dominant cause of non-tumoral HPRL. When the origin is pituitary, there is a predominance of late-diagnosed macroadenomas.

These peculiarities demand a high index of suspicion. The diagnostic approach must systematically begin with medication reconciliation and the rigorous exclusion of systemic causes and analytical artifacts, such as MPRL, before resorting to MRI. Therapeutic management requires a tailored approach, prioritizing the individualization of dopamine agonist dosing and careful consideration of risks in this vulnerable population.
